# The effect of replacing egg yolk with sesame–peanut defatted meal milk on the physicochemical, colorimetry, and rheological properties of low‐cholesterol mayonnaise

**DOI:** 10.1002/fsn3.616

**Published:** 2018-03-14

**Authors:** Mahsa Karshenas, Mohammad Goli, Nafiseh Zamindar

**Affiliations:** ^1^ Department of Food Science and Technology Isfahan (Khorasgan) Branch Islamic Azad University Isfahan Iran

**Keywords:** colorimetry, egg yolk, mayonnaise, physicochemical properties, rheological properties, sesame–peanut meal milk

## Abstract

Egg yolk was replaced with sesame–peanut meal milk in mayonnaise in the levels of 0, 25%, 50%, 75%, and 100%. The pH was significantly decreased by increasing the percentage of replacement in all three kinds of replacement (*p* < .05). However, over the whole period, no significant difference was observed in the acidity. The mayonnaise samples, except for the replacements of 50%, were desirable in terms of physical and thermal stability. A significant decrease was seen in lightness (L*) and yellowness (b*) of the samples as a result of an increase in the percentage of replacements (*p* < .05). In the power law model, the flowing index amount (*n*) of all samples was in the domain between zero and one, which served as evidence for pseudoplastic behavior (dilatant with shear) of mayonnaise samples. The positive results are employing suitably the sesame–peanut meal milk instead of egg yolk, decreasing the cholesterol of mayonnaise and increasing its nutritional value, proposing mayonnaise factories to make use of the meal of the oil extraction factory as emulsifier, which lead to a decrease in overall costs of producing these products.

## 1 INTRODUCTION

**Figure 1:**
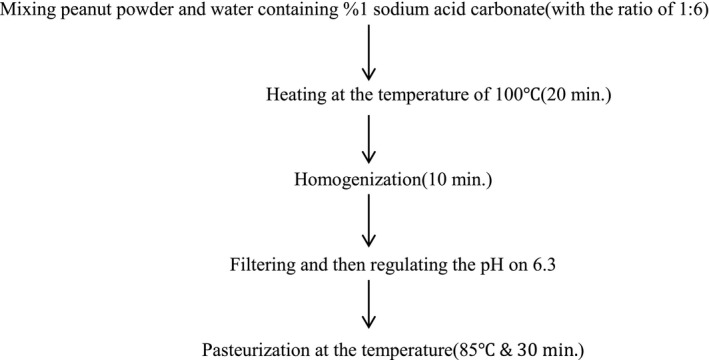
Flow diagram for the preparation of peanut meal milk

**Figure 2:**
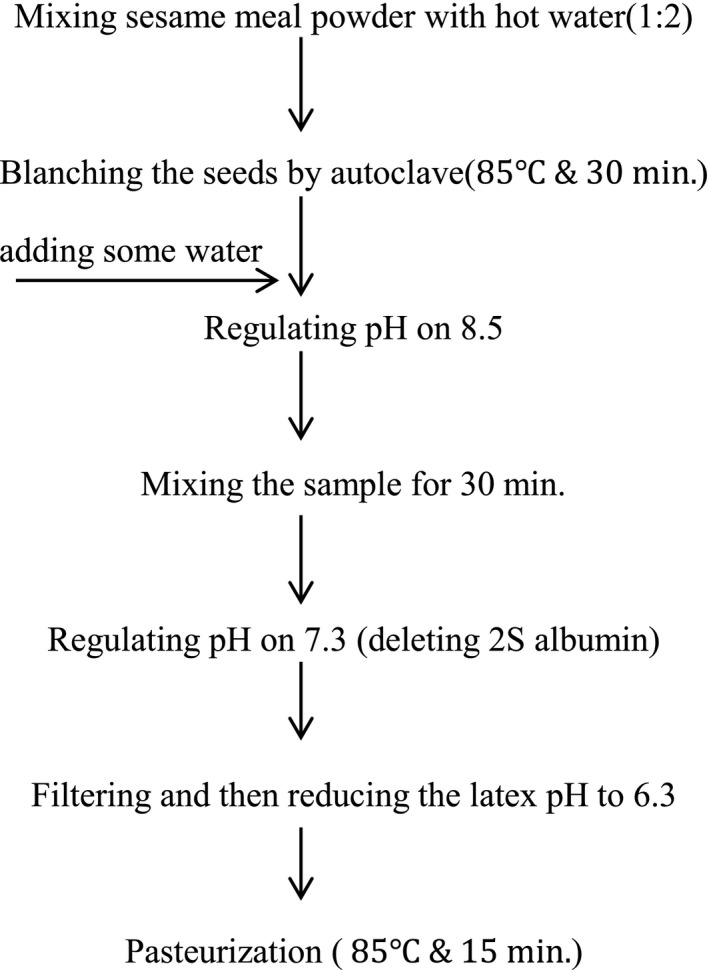
Flow diagram for the preparation of sesame meal milk

Nowadays, the use of food industrial waste for nutritional purposes, that is, orange juice enriched with encapsulated polyphenolic extract of lime waste (Afkhami, Goli, & Keramat, 2017), or functional purposes, that is, substitution of sesame and peanut meal milk for egg yolk in the low‐cholesterol mayonnaise to improve rheological properties and/or oak flour as a replacement of wheat and corn flour to improve the antioxidant activity in biscuit (Parsaei, Goli, & Abbasi, 2018), has become widespread. Mayonnaise is a kind of emulsion of oil in water in which the drops of oil are sporadic as dispersed phase within the aqueous phase, including vinegar. In the emulsion system of mayonnaise, the protein and lipoprotein molecules in the egg form a layer around the drops of oil and prevent them concocted with each other and thus making the content instable (Goankar, Rathna, Chen, & Campbell, 2010). Despite the fact that egg in mayonnaise plays significant roles such as emulsification and determining the taste and color of the product, due to the large amounts of cholesterol that which results in cardiovascular diseases (Anton et al., 2003), hesitancy in consuming this spicy food is prevalent. Therefore, a large number of studies have been carried out on the possibility of the egg's removal and its replacement with something else in mayonnaise. In this regard, the emulsifier activity of the soya, wheat, and milk proteins has been already investigated by the former researchers (Abu‐ghoush, Samhouri, Al‐Holy, & Herald, 2008; Goankar et al., 2010). Sesame seed contains about 20%–25% protein, and the defatted sesame meal milk is rich in terms of protein, methionine, calcium, phosphorous, and niacin (El‐Adawy, 1997; Rir, Feldman, Aserin, & Garti, 1994). According to Pastorello et al. (2001), one of the most important allergic substances in sesame is a protein with the molecular weight of about 9 kDa. The investigation of the sequence of amino acids in the allergic protein indicated that this material is a 2s‐albumin and is in the category of cereal alpha‐amylase and trypsin inhibitors (Khalid & Babiker, 2003; Wolff et al., 2003, 2004). One of the protein products of sesame is a milky extract, which is called sesame milk. The sesame meal milk contains more proteins compared with the sesame seed milk. This is probably because of the effect of the process of oil extraction by pressing as the cell walls are destroyed under the above pressure, and the protein is more likely to exit during the process of extraction. Peanut contains 22%–30% protein rich in arginine and is considered to be one of the most important sources of protein in the world (Jianmei, Ahmedna, & Goktepe, 2007; Jianmei, Ahmedna, Goktepe, Cheng, & Maleki, 2011). Consuming raw peanut or other foods containing raw peanut can create allergen reaction in some people (Sampson, 2004). Although sesame and peanut meal contains allergic protein 2s‐albumin, it can be easily removed by various pH (7.3)/temperature (85–100°C)/time (20–30 min) treatments according to methods shown in Figures 1 and 2 (Comstock, Maleki, & Teuber, 2016). One of the foods obtained from peanut is the peanut milk, which can be produced out of raw and fatty peanut, roasted peanut, peanut flour without fat or with a tiny amount of fat (Chan & Beuchat, 1991). These practical features, such as emulsifier activities in the protein of sesame (Chan & Beuchat, 1991) and peanut (Wuh, Ma, & Ren, 2009), have already been investigated by researchers. Due to the emulsifying role of the sesame and peanut meal milk, which is a suitable substitute for egg yolk in the low‐cholesterol mayonnaise, advantages such as consumer health benefits—protein source rich in arginine and the other essential amino acids with high nutritional value, and economical benefits for oil extraction factories—using their wastes as a by‐product with high added value can be reaped, whereas, the conventional mayonnaise does not have fiber materials. Therefore, the substitution of egg yolk with peanut and sesame meal milk can remove this deficiency and improve the nutritional and functional properties of mayonnaise and the other foodstuffs. This study aimed at examining the capability of using sesame–peanut meal (as a by‐product in oil extraction factories) as replacement for yolk in production of low‐cholesterol mayonnaise with high nutritional value (Parsaei et al., 2018) and low cost of production, as well as reviewing the physicochemical features (i.e., pH, acidity, physical and thermal stability, and the color parameters) of resulted mayonnaise during the period of 2‐month storage at the temperature of 25 ± 1°C.

## 2 MATERIALS AND METHODS

### 2.1 Peanut meal milk preparation

For producing peanut meal milk, the method of Salunkhe and Kadam (1989) was employed with some changes. Peanut meal powder was mixed with the water containing 1% sodium bicarbonate (1:6). Then, it was heated for 20 min at the temperature of 100°C. After 10‐min homogenization, in order to obtain its latex, filtering was performed using a filter paper. The latex's pH was reduced to 6.3 by the 0.5 N acid citric. After a 10‐min homogenization by blender (Molineux, France) at the temperature of 85°C, the pasteurization of the peanut meal milk was carried out for 30 min. The method of producing peanut meal milk is shown in the flowchart of Figure 1.

### 2.2 Sesame meal milk preparation

The method of producing sesame meal milk is indicated in the flowchart of Figure 2. First, sesame meal powder was mixed with hot water with a ratio of 1 to 2, and blanching was performed using autoclave at the temperature of 100°C for 30 min. Then, some water was added to the mixture, and its pH was increased to 8.5 for the sake of better extraction of the proteins in water. After mixing the sample by blender for 30 min (Molineux, France), pH was reduced to 7.3 for removing 2s‐albumin because of its allergic feature. Finally, the latex was separated from the other materials using a filter paper and sesame meal milk pH was set at 6.3 and was pasteurized at the temperature of 100°C (Khodaparast, Najafi, Elhami‐Rad, & Divandari, 2005).

### 2.3 Mayonnaise preparation

In order to produce mayonnaise, the formula included the following—sunflower oil: 60%; emulsifier: 12%; water: 13.5%; vinegar: 8.825%; sugar: 4%; mustard: 0.4%; salt: 0.7%; acid citric: 0.1%; sodium benzoate: 0.075%; and guar:xanthan ratio: (0.2:0.2)%. Different ratios of egg replacement with sesame, peanut meal milk, and a mixture of these (50:50) were made in the levels of 0%, 25%, 50%, 75%, and 100%. First, the powder materials of the formula (sugar, salt, mustard, acid citric, and sodium benzoate) accompanied by emulsifier were mixed for 4 min in the mixer with the speed round of 800, then, half of the oil, which was already mixed with gums, was added to the mixture in the mixer with the speed round of 1000, and then, the rest of the oil was added to the mixture. After that, vinegar was gradually added to the mixture, and finally, the mayonnaise was homogenized in the mixer with the speed round of 1,500 for 7 min. The samples were kept at the temperature of 25 ± 1°C for 2 months, and the physicochemical and colorimetry tests were performed on the samples in the first, 20th, 30th, 40th, and 60th days after production.

### 2.4 The pH and acidity measurement

pH measurements of the mayonnaise samples were taken using pH meter (Model 3510; Bibby Scientific Limited, UK) at the temperature of ~25°C, and the acidity measurement of the samples was taken by titration with 0.1 N in the presence of the phenolphthalein reagent for three times.

### 2.5 Stability tests

In order to measure the physical stability, 15 g (F_0_) of the sample was taken in the centrifuge tubes with specific weight, and the tubes were centrifuged for 30 min at a speed of 500 rpm. After this stage, the layer of oil was removed and the weight of the sediment was measured (F_1_). This test was performed three times, and the emulsion stability was calculated according to the percentage using formula 1 (Mun et al., 2009):

(1) $$\text{The percentage of physical stability}=\frac{F_{1}}{F_{0}}\times 100.$$

In order to determine the thermal stability, 15 g of the sample was taken in the centrifuge tubes, the samples were placed at the temperature of 50°C for 48 hr, and then, they were centrifuged at a speed of 3000 rpm for 10 min. The layer of oil was removed after the centrifugation, and the sediment weight was measured and calculated using Equation (1), the percentage of thermal stability (Mun et al., 2009).

### 2.6 Color measurement

Colorimetry test was performed in the first, 20th, 40th, and 60th days after production. In order to determine the color indexes of the mayonnaise samples, the hunter laboratory colorimeter was used. L* indicates the lightness and ranges from 0 to 100, a* indicates redness (redness: + and greenness: −), and b* indicates the ellowness (yellow: + and blue: −) whose ranges differ from −120 to +120. Furthermore, the overall color difference (Δ*Ε*) was calculated using Equation (2) between the first and sixtieth days of storage.

(2) $$\Delta E=\sqrt{{\Delta L}^{*2}+{\Delta a}^{*2}+{\Delta b}^{*2}}.$$

### 2.7 Texture profile analysis

The textural properties of mayonnaise samples were studied with the extrusion press test using a device for the evaluation of texture properties (LFRA 4500; Brookfield, USA) the day after production. This test involves a cylindrical probe with specified geometry inside the standard dish. Parameters such as hardness, adhesiveness, and adhesive force were calculated with this test. The hardness, which reaches the highest point of the diagram at the time (maximum point of the diagram), can be stated in Newton or gram unit. The adhesive force is the force necessary to separate the probe from the sample. It is also the most negative force produced by the probe while returning and exiting from the measurement dish in gram or Newton unit (minimum point of the diagram). Adhesiveness, in Newton.second (N.s) or gram.second (g.s), is the negative area of the diagram and is a symbol of the product texture cohesion (Khodaparast et al., 2005). In order to do this test, a probe with a diameter of 38 mm, penetration amount of 20 mm, and penetration speed of 1 mm/s was applied. A metal cylinder with an internal diameter of 45 mm and height of 95 mm was selected for this test. About 70 g of sample was weighed for the test and was poured into the cylinder. The measurements were taken in three replications 24 hours after the production of samples.

### 2.8 Statistical analysis

All tests were conducted in a completely randomized design with three replications using the SAS 9.0 software (Inst. Inc., Cary, NC, USA). All statistical comparisons were performed using Duncan's test at the level of 5%.

## 3 RESULTS AND DISCUSSION

### 3.1 Comparing the pH of mayonnaise samples during of the storage period

In Table 1, the comparisons between the pH of different treatments of mayonnaise were made during each period of storage. According to the results reported, there was a significant difference between the pH of the treatments in each time of the period (*p* < .05). Also, the results of investigating the pH over a 2‐month period of storage indicate a significant difference in the levels of equal replacement (*p* < .05) (Table 1). The trend of changes in pH from the beginning to the end of the period has been downward in all the samples. With regard to Table 1, the pH of all the samples in the 60th day was significantly lower than the day after production. The decrease in pH was probably due to the breaking of some ester groups (triglycerides and phospholipids within oil and egg yolk in mayonnaise formula) and their change into acid groups (Bostani, Ahmed, & Salem, 2011; Karas, Skuarca, & Zlender, 2002). As shown in Table 2, the replaced samples of the peanut meal milk bear a close resemblance to the control sample in terms of the mean of pH during the whole 2‐month period compared with the other two kinds of replacements. No significant difference was observed between the control sample and replaced samples until 75% replacement. The highest pH mean belonged to control sample and P25, and the lowest pH mean belonged to the samples of S75 and S100. pH mean in samples during the preservation period decreased with increasing the percentage of replacement so that the pH of the 100% samples in all three kinds of replacements (peanut, sesame, peanut–sesame meal milk), and there was a significant difference with the control sample. Probably, this is due to the effect of pH of meal milk replaced with egg in the final product. With regard to the fact that the environment of mayonnaise is not in the buffer mode, the type of meal milk could have influenced the pH of the mayonnaise and also could be attributed to greater water content in mayonnaises that contained sesame and peanut, especially in higher replacement levels, which might improve the growth of acid‐tolerant microorganisms such as lactic acid bacteria (Bostani et al., 2011; Karas et al., 2002).

**Table 1:** Comparison of mayonnaise pH (in all replacement levels of egg yolk with meal milk) at any time from 60‐day period in storage temperature (25 ± 1°C)

Treatment	pH				
	Day				
	1	20	30	40	60
Blank	$4.09\pm 0.02_{A}^{a}$	$4.06\pm 0.02_{A}^{a}$	$4.02\pm 0.02_{B}^{a}$	$4.01\pm 0.01_{B}^{\mathrm{ab}}$	$3.85\pm 0.02_{C}^{a}$
25 Peanut–Sesame	$4.07\pm 0.02_{A}^{\mathrm{abc}}$	$4.05\pm 0.04_{\mathrm{AB}}^{\mathrm{ab}}$	$4\pm 0.01_{\mathrm{BC}}^{\mathrm{ab}}$	$3.97\pm 0.03_{C}^{\mathrm{bc}}$	$3.79\pm 0.04_{D}^{\mathrm{abc}}$
50 Peanut–Sesame	$4.02\pm 0.02_{A}^{\mathrm{cdef}}$	$4.01\pm 0.01_{\mathrm{AB}}^{\mathrm{ab}}$	$3.99\pm 0.02_{\mathrm{AB}}^{\mathrm{ab}}$	$3.96\pm 0.01_{B}^{\mathrm{cd}}$	$3.75\pm 0.06_{C}^{\mathrm{cde}}$
75 Peanut–Sesame	$4.01\pm 0.06_{A}^{\mathrm{def}}$	$3.98\pm 0.05_{\mathrm{AB}}^{\mathrm{bc}}$	$3.93\pm 0.2_{\mathrm{AB}}^{\mathrm{cd}}$	$3.92\pm 0.02_{B}^{\mathrm{ef}}$	$3.73\pm 0.06_{C}^{\mathrm{cde}}$
100 Peanut–Sesame	$3.94\pm 0.02_{A}^{g}$	$3.85\pm 0.09_{\mathrm{AB}}^{d}$	$3.83\pm 0.06_{B}^{e}$	$3.82\pm 0.05_{B}^{g}$	$3.66\pm 0.01_{C}^{\mathrm{ef}}$
25 Peanut	$4.07\pm 0.02_{A}^{\mathrm{ab}}$	$4.05\pm 0.03_{\mathrm{AB}}^{a}$	$4.02\pm 0.01_{B}^{a}$	$4.02\pm 0.02_{B}^{a}$	$3.83\pm 0.02_{C}^{\mathrm{ab}}$
50 Peanut	$4.06\pm 0.03_{A}^{\mathrm{abcd}}$	$4.05\pm 0.04_{\mathrm{AB}}^{\mathrm{ab}}$	$4.02\pm 0.00_{\mathrm{AB}}^{\mathrm{ab}}$	$4.00\pm 0.01_{B}^{\mathrm{abc}}$	$3.78\pm 0.01_{C}^{\mathrm{bcd}}$
75 Peanut	$4.06\pm 0.03_{A}^{\mathrm{abcde}}$	$4.04\pm 0.03_{\mathrm{AB}}^{\mathrm{abc}}$	$4.02\pm 0.02_{\mathrm{AB}}^{a}$	$3.99\pm 0.02_{B}^{\mathrm{abc}}$	$3.78\pm 0.04_{C}^{\mathrm{abcd}}$
100 Peanut	$4.00\pm 0.01_{A}^{\mathrm{ef}}$	$3.99\pm 0.02_{A}^{a}$	$3.93\pm 0.03_{B}^{\mathrm{cd}}$	$3.93\pm 0.02_{B}^{\mathrm{de}}$	$3.72\pm 0.03_{C}^{\mathrm{cde}}$
25 Sesame	$4.03\pm 0.02_{A}^{\mathrm{bcde}}$	$4.00\pm 0.03_{\mathrm{AB}}^{\mathrm{ab}}$	$3.97\pm 0.2_{B}^{\mathrm{bc}}$	$3.97\pm 0.03_{B}^{c}$	$3.76\pm 0.05_{C}^{\mathrm{bcd}}$
50 Sesame	$3.98\pm 0.04_{A}^{\mathrm{fg}}$	$3.93\pm 0.02_{\mathrm{AB}}^{c}$	$3.89\pm 0.03_{B}^{d}$	$3.87\pm 0.03_{B}^{f}$	$3.71\pm 0.04_{C}^{\mathrm{de}}$
75 Sesame	$3.86\pm 0.03_{A}^{h}$	$3.85\pm 0.05_{A}^{d}$	$3.83\pm 0.02_{\mathrm{AB}}^{\mathrm{ef}}$	$3.78\pm 0.01_{B}^{g}$	$3.63\pm 0.04_{C}^{f}$
100 Sesame	$3.82\pm 0.04_{A}^{h}$	$3.81\pm 0.01_{A}^{d}$	$3.78\pm 0.02_{A}^{f}$	$3.70\pm 0.02_{B}^{h}$	$3.62\pm 0.02_{C}^{f}$

Assays were performed in triplicate. Mean ± SD values, followed by the same superscript letter within each column (day) and the same subscript letter within each row (treatment), have no significant differences in *p* ≤ .05 by ANOVA.

**Table 2:** Comparison of mayonnaise pH, acidity, physical stability, thermal stability, and ∆E (in all replacement levels of egg yolk with meal milk) at total 60‐day period in storage temperature (25 ± 1°C)

Treatment	pH	Acidity (g acetic acid/100 g)	Physical stability (%)	Thermal stability (%)	∆E (60th→1st day)
Blank	4 ± 0.09^a^	0.62 ± 0.02^b^	99.89 ± 0.25^a^	99.69 ± 0.37^a^	2.13 ± 0.24^a^
25 Peanut–Sesame	3.98 ± 0.1^ab^	0.62 ± 0.02^b^	99.75 ± 0.43^ab^	99.47 ± 0.53^abc^	4.03 ± 0.69^abc^
50 Peanut–Sesame	3.95 ± 0.1^abc^	0.63 ± 0.02^ab^	98.63 ± 0.02^d^	98.37 ± 2.25^d^	4.45 ± 0.77^abc^
75 Peanut–Sesame	3.91 ± 0.11^bc^	0.63 ± 0.02^ab^	99.77 ± 0.38^ab^	99.54 ± 0.44^ab^	5.24 ± 2.6^a^
100 Peanut–Sesame	3.82 ± 0.1^de^	0.64 ± 0.1^ab^	99.9 ± 0.25^a^	99.77 ± 0.32^a^	3.2 ± 1.99^abc^
25 Peanut	4 ± 0.09^a^	0.62 ± 0.02^b^	99.67 ± 0.53^abc^	99.4 ± 0.56^abc^	2.63 ± 1.07^bc^
50 Peanut	3.98 ± 0.11^ab^	0.63 ± 0.02^ab^	99.11 ± 1.55^bcd^	98.68 ± 1.69^bcd^	3.38 ± 1.13^abc^
75 Peanut	3.98 ± 0.11^ab^	0.63 ± 0.02^ab^	99.77 ± 0.37^ab^	99.43 ± 0.54^abc^	4.58 ± 0.77^abc^
100 Peanut	3.91 ± 0.12^bc^	0.64 ± 0.02^ab^	99.95 ± 0.12^a^	99.72 ± 0.37^a^	3.68 ± 0.52^abc^
25 Sesame	3.95 ± 0.1^abc^	0.63 ± 0.02^ab^	99.64 ± 0.63^abc^	99.34 ± 0.73^abc^	3.1 ± 0.87^abc^
50 Sesame	3.88 ± 0.1^cd^	0.63 ± 0.02^ab^	99.00 ± 1.49^cd^	98.61 ± 2.09^cd^	2.63 ± 1.08^bc^
75 Sesame	3.79 ± 0.09^e^	0.64 ± 0.02^ab^	99.78 ± 0.37^ab^	99.43 ± 0.62^abc^	3.06 ± 0.19^abc^
100 Sesame	3.75 ± 0.08^e^	0.64 ± 0.02^ab^	99.98 ± 0.07^a^	99.77 ± 0.29^a^	4.7 ± 0.13^ab^

Mean ± SD values, followed by the same superscript letter within each column, are not significant differences in *p* ≤ .05 by ANOVA.

### 3.2 Comparing the acidity of mayonnaise samples during the storage period

Among the acidity of different samples of mayonnaise, the difference in each time of storage was significant in the first to 30th days (*p* < .05). However, in the 40th day, no significant difference was observed among the treatments (*p* > .05), and in the last day of the 2‐month period, the difference in samples was once again significant in terms of acidity (*p* < .05) (Table 3). The results of comparing acidity during the storage period recommend a significant difference in the level of equal replacement (*p* < .05), and the trend of changes in acidity during the storage period was upward for all the samples in the 60th and first days (Table 3), which was in line with the results of other researchers (Bostani et al., 2011; Karas et al., 2002). Probably, breaking the ester groups (triglycerides and phospholipids within oil and egg yolk in mayonnaise formula) and their changing into acid groups can also be influential in increasing the acidity of samples and, on the other hand, the growth of microorganisms, which are resistant to the acidic conditions, the same as lactic acid bacteria, can lead to an increase in acidity. In sum, there was no significant difference between the acidity mean during the whole period of storage for control sample and the other samples (*p* > .05) (Table 2).

**Table 3:** Comparison of the mayonnaise acidity (in all replacement levels of egg yolk with meal milk) at any time from 60‐day period in storage temperature (25 ± 1°C)

Treatment	Acidity (g acetic acid/100 g)				
	Days				
	1	20	30	40	60
Blank	$0.6\pm 0.00_{D}^{b}$	$0.61\pm 0.01_{\mathrm{CD}}^{\mathrm{bc}}$	$0.62\pm 0.01_{\mathrm{BC}}^{\mathrm{bc}}$	$0.63\pm 0.01_{\mathrm{AB}}^{a}$	$0.65\pm 0.01_{A}^{c}$
25 Peanut–Sesame	$0.61\pm 0.00_{D}^{b}$	$0.61\pm 0.01_{\mathrm{CD}}^{\mathrm{bc}}$	$0.61\pm 0.00_{C}^{c}$	$0.64\pm 0.01_{B}^{a}$	$0.65\pm 0.00_{A}^{\mathrm{bc}}$
50 Peanut–Sesame	$0.62\pm 0.00_{C}^{a}$	$0.62\pm 0.01_{C}^{\mathrm{abc}}$	$0.63\pm 0.01_{B}^{\mathrm{ab}}$	$0.64\pm 0.00_{B}^{a}$	$0.65\pm 0.01_{A}^{\mathrm{abc}}$
75 Peanut–Sesame	$0.62\pm 0.00_{C}^{a}$	$0.62\pm 0.01_{C}^{\mathrm{abc}}$	$0.63\pm 0.01_{\mathrm{BC}}^{\mathrm{ab}}$	$0.64\pm 0.00_{B}^{a}$	$0.66\pm 0.00_{A}^{\mathrm{abc}}$
100 Peanut–Sesame	$0.62\pm 0.00_{D}^{a}$	$0.62\pm 0.00_{\mathrm{CD}}^{a}$	$0.63\pm 0.01_{\mathrm{BC}}^{\mathrm{ab}}$	$0.64\pm 0.00_{B}^{a}$	$0.66\pm 0.00_{A}^{\mathrm{ab}}$
25 Peanut	$0.61\pm 0.00_{D}^{b}$	$0.61\pm 0.01_{D}^{c}$	$0.62\pm 0.01_{C}^{\mathrm{bc}}$	$0.64\pm 0.00_{B}^{a}$	$0.65\pm 0.01_{A}^{c}$
50 Peanut	$0.61\pm 0.00_{E}^{b}$	$0.62\pm 0.00_{D}^{\mathrm{abc}}$	$0.63\pm 0.01_{C}^{\mathrm{ab}}$	$0.64\pm 0.01_{B}^{a}$	$0.65\pm 0.00_{A}^{\mathrm{bc}}$
75 Peanut	$0.61\pm 0.00_{B}^{a}$	$0.62\pm 0.01_{B}^{\mathrm{ab}}$	$0.63\pm 0.00_{B}^{\mathrm{ab}}$	$0.64\pm 0.01_{\mathrm{AB}}^{a}$	$0.65\pm 0.02_{A}^{\mathrm{bc}}$
100 Peanut	$0.62\pm 0.01_{C}^{a}$	$0.62\pm 0.00_{C}^{\mathrm{ab}}$	$0.63\pm 0.00_{B}^{\mathrm{ab}}$	$0.64\pm 0.00_{B}^{a}$	$0.66\pm 0.00_{A}^{\mathrm{abc}}$
25 Sesame	$0.62\pm 0.00_{C}^{a}$	$0.62\pm 0.01_{C}^{\mathrm{abc}}$	$0.63\pm 0.01_{B}^{\mathrm{ab}}$	$0.64\pm 0.00_{B}^{a}$	$0.66\pm 0.00_{A}^{\mathrm{abc}}$
50 Sesame	$0.62\pm 0.00_{C}^{a}$	$0.62\pm 0.00_{C}^{\mathrm{ab}}$	$0.63\pm 0.00_{\mathrm{BC}}^{\mathrm{ab}}$	$0.64\pm 0.01_{B}^{a}$	$0.66\pm 0.01_{A}^{\mathrm{abc}}$
75 Sesame	$0.62\pm 0.00_{D}^{a}$	$0.62\pm 0.01_{\mathrm{CD}}^{a}$	$0.63\pm 0.01_{\mathrm{BC}}^{\mathrm{ab}}$	$0.64\pm 0.01_{B}^{a}$	$0.67\pm 0.01_{A}^{a}$
100 Sesame	$0.62\pm 0.00_{C}^{a}$	$0.63\pm 0.01_{C}^{a}$	$0.64\pm 0.01_{B}^{a}$	$0.65\pm 0.00_{B}^{a}$	$0.67\pm 0.00_{A}^{a}$

Assays were performed in triplicate. Mean ± SD values, followed by the same superscript letter within each column (day) and the same subscript letter within each row (treatment), have not significant differences in *p* ≤ .05 by ANOVA.

### 3.3 Comparing the stability of mayonnaise samples during the storage period

Stable emulsion refers to an emulsion in which sedimentation did not occur. After examining the trend of changes in physical stability during 2 months, we realized that the effect of time on the samples’ physical stability has been decreasing the stability. The physical stability percentage of samples was 100 until the 20th day, and since the 30th to 60th days, a decrease was observed. Furthermore, a significant difference in the physical stability among the samples was observed since the half of the period of storage (the 30th day) till the end of it (*p* < .05) (Table 4). Furthermore, the results of physical stability suggest a significant difference between the samples with the equal levels of replacement (*p* < .05) (Table 4). The lowest mean was observed in the samples of 50%, in which no significant difference was observed in the means of physical stability among the 50% samples in all three kinds of replacements. The highest mean of physical stability belonged to the 100% replaced samples (without egg) and the control sample (Table 2). With regard to the results of Table 5 in the day after production, all the samples were of 100% thermal stability and in the 20th day, a little decrease was observed in the thermal stability of the 50% treatment in all three kinds of replacements. However, these differences among the samples were not significant (*p* > .05). In the 30th, 40th, and 60th days, there was a significant difference between the samples’ thermal stability (*p* < .05). The results of thermal stability changes, the same as physical stability during the period of storage, indicate a significant difference in the levels with the equal percentage of replacement (*p* < .05) (Table 5). The lowest thermal stability was observed in the samples of 50% replacement in which no significant difference was observed between their thermal stability means. The highest thermal stability also belonged to the control samples and 100% replaced samples (without egg) (Table 2). Having a higher physical and thermal stability of 100% samples replaced with sesame–peanut, peanut, and sesame meal milk instead of egg compared with other samples, stood in contrast with the results of the study carried out by Mun et al. (2009). It was also in contrast with Stock's law based on the direct relationship between increasing viscosity and stability. Probably, this originates from the high emulsion power of the meals’ protein used in the study. The samples of 50% replacements were of the lowest level of stability among the other samples. The reason for this can be attributed to the antagonist effect of egg protein and the protein present in the meal milk (Herald, Abu‐goush, & Aramoun, 2009).

**Table 4:** Comparison of the mayonnaise physical stability (in all replacement levels of egg yolk with meal milk) at any time from 60‐day period in storage temperature (25 ± 1°C)

Treatment	Physical stability (%)				
	Days				
	1	20	30	40	60
Blank	$100\pm 0.00_{A}^{a}$	$100\pm 0.00_{A}^{a}$	$100\pm 0.00_{A}^{a}$	$99.91\pm 0.1_{A}^{a}$	$99.53\pm 0.41_{B}^{a}$
25 Peanut–Sesame	$100\pm 0.00_{A}^{a}$	$100\pm 0.00_{A}^{a}$	$100\pm 0.00_{A}^{a}$	$99.96\pm 0.44_{A}^{a}$	$99.1\pm 0.39_{B}^{a}$
50 Peanut–Sesame	$100\pm 0.00_{A}^{a}$	$100\pm 0.00_{A}^{a}$	$99.78\pm 0.21_{A}^{b}$	$98.46\pm 0.47_{B}^{b}$	$98.82\pm 0.4_{C}^{c}$
75 Peanut–Sesame	$100\pm 0.00_{A}^{a}$	$100\pm 0.00_{A}^{a}$	$100\pm 0.00_{A}^{a}$	$99.71\pm 0.1_{A}^{a}$	$99.13\pm 0.4_{B}^{a}$
100 Peanut–Sesame	$100\pm 0.00_{A}^{a}$	$100\pm 0.00_{A}^{a}$	$100\pm 0.00_{A}^{a}$	$99.84\pm 0.27_{A}^{a}$	$99.64\pm 0.45_{A}^{a}$
25 Peanut	$100\pm 0.00_{A}^{a}$	$100\pm 0.00_{A}^{a}$	$100\pm 0.00_{A}^{a}$	$99.56\pm 0.43_{A}^{a}$	$98.82\pm 0.45_{B}^{a}$
50 Peanut	$100\pm 0.00_{A}^{a}$	$100\pm 0.00_{A}^{a}$	$100\pm 0.00_{A}^{b}$	$98.96\pm 0.32_{A}^{b}$	$96.87\pm 2.46_{B}^{b}$
75 Peanut	$100\pm 0.00_{A}^{a}$	$100\pm 0.00_{A}^{a}$	$100\pm 0.00_{A}^{a}$	$99.63\pm 0.21_{A}^{a}$	$99.24\pm 0.41_{B}^{a}$
100 Peanut	$100\pm 0.00_{A}^{a}$	$100\pm 0.00_{A}^{a}$	$100\pm 0.00_{A}^{a}$	$99.96\pm 0.01_{A}^{a}$	$99.8\pm 0.24_{A}^{a}$
25 Sesame	$100\pm 0.00_{A}^{a}$	$100\pm 0.00_{A}^{a}$	$100\pm 0.00_{A}^{a}$	$99.67\pm 0.12_{A}^{a}$	$98.51\pm 0.54_{B}^{a}$
50 Sesame	$100\pm 0.00_{A}^{a}$	$100\pm 0.00_{A}^{a}$	$100\pm 0.00_{A}^{b}$	$98.77\pm 0.25_{B}^{b}$	$96.38\pm 0.99_{C}^{b}$
75 Sesame	$100\pm 0.00_{A}^{a}$	$100\pm 0.00_{A}^{a}$	$100\pm 0.00_{A}^{a}$	$99.75\pm 0.32_{A}^{a}$	$99.13\pm 0.11_{B}^{a}$
100 Sesame	$100\pm 0.00_{A}^{a}$	$100\pm 0.00_{A}^{a}$	$100\pm 0.00_{A}^{a}$	$99.98\pm 0.04_{A}^{a}$	$99.91\pm 0.15_{A}^{a}$

Assays were performed in triplicate. Mean ± SD values, followed by the same superscript letter within each column (day) and the same subscript letter within each row (treatment), have no significant differences in *p* ≤ .05 by ANOVA.

**Table 5:** Comparison of the mayonnaise thermal stability (in all replacement levels of egg yolk with meal milk) at any time from 60‐day period in storage temperature (25 ± 1°C)

Treatment	Thermal stability (%)				
	Days				
	1	20	30	40	60
Blank	$100\pm 0.00_{A}^{a}$	$100\pm 0.00_{A}^{a}$	$99.89\pm 0.02_{A}^{\mathrm{ab}}$	$99.38\pm 0.19_{B}^{\mathrm{abc}}$	$99.2\pm 0.18_{B}^{a}$
25 Peanut–Sesame	$100\pm 0.00_{A}^{a}$	$100\pm 0.00_{A}^{a}$	$99.47\pm 0.17_{B}^{c}$	$99.18\pm 0.1_{B}^{\mathrm{abcd}}$	$98.71\pm 0.3_{C}^{a}$
50 Peanut–Sesame	$100\pm 0.00_{A}^{a}$	$99.96\pm 0.01_{A}^{a}$	$99.25\pm 0.35_{\mathrm{AB}}^{c}$	$98.16\pm 0.34_{B}^{f}$	$94.4\pm 1.38_{C}^{b}$
75 Peanut–Sesame	$100\pm 0.00_{A}^{a}$	$100\pm 0.00_{A}^{a}$	$99.45\pm 0.2_{B}^{c}$	$99.19\pm 0.2_{\mathrm{BC}}^{\mathrm{abcd}}$	$99.07\pm 0.35_{C}^{a}$
100 Peanut–Sesame	$100\pm 0.00_{A}^{a}$	$100\pm 0.00_{A}^{a}$	$99.91\pm 0.15_{A}^{\mathrm{ab}}$	$99.64\pm 0.37_{A}^{a}$	$99.29\pm 0.14_{B}^{a}$
25 Peanut	$100\pm 0.00_{A}^{a}$	$100\pm 0.00_{A}^{a}$	$99.24\pm 0.21_{B}^{c}$	$99.07\pm 0.07_{\mathrm{BC}}^{\mathrm{bcd}}$	$98.71\pm 0.38_{C}^{a}$
50 Peanut	$100\pm 0.00_{A}^{a}$	$100\pm 0.00_{B}^{a}$	$99.33\pm 0.27_{C}^{c}$	$98.58\pm 0.21_{D}^{\mathrm{ef}}$	$95.62\pm 0.59_{E}^{b}$
75 Peanut	$100\pm 0.00_{A}^{a}$	$100\pm 0.00_{A}^{a}$	$99.31\pm 0.38_{B}^{c}$	$98.87\pm 0.23_{C}^{\mathrm{de}}$	$98.96\pm 0.14_{\mathrm{BC}}^{a}$
100 Peanut	$100\pm 0.00_{A}^{a}$	$100\pm 0.00_{A}^{a}$	$99.91\pm 0.15_{B}^{\mathrm{ab}}$	$99.44\pm 0.41_{A}^{\mathrm{abc}}$	$99.25\pm 0.15_{B}^{a}$
25 Sesame	$100\pm 0.00_{A}^{a}$	$100\pm 0.00_{A}^{a}$	$99.53\pm 0.12_{B}^{\mathrm{bc}}$	$99.00\pm 0.18_{C}^{\mathrm{cde}}$	$98.16\pm 0.1_{D}^{a}$
50 Sesame	$100\pm 0.00_{A}^{a}$	$99.98\pm 0.00_{A}^{a}$	$99.38\pm 0.25_{A}^{c}$	$98.18\pm 0.31_{A}^{a}$	$95.52\pm 3.03_{B}^{b}$
75 Sesame	$100\pm 0.00_{A}^{a}$	$100\pm 0.00_{A}^{a}$	$99.56\pm 0.14_{B}^{\mathrm{abc}}$	$99.16\pm 0.33_{C}^{\mathrm{abcd}}$	$98.45\pm 0.2_{D}^{a}$
100 Sesame	$100\pm 0.00_{A}^{a}$	$100\pm 0.00_{A}^{a}$	$99.96\pm 0.01_{A}^{a}$	$99.53\pm 0.13_{B}^{\mathrm{ab}}$	$99.35\pm 0.08_{C}^{a}$

Assays were performed in triplicate. Mean ± SD values, followed by the same superscript letter within each column (day) and the same subscript letter within each row (treatment), have no significant differences in *p* ≤ .05 by ANOVA.

### 3.4 Comparing the color parameters obtained from the mayonnaise samples during the storage period

Mayonnaise lightness (L*) is one of the important factors of acceptance among the consumers. The comparison between different treatments in each time of storage suggested a significant difference between the lightness of different samples (*p* < .05) (Table 6). Sample P‐S25 was of the highest level of lightness compared with the other samples over the period of storage, and sample S100 had the lowest level of lightness over the period of storage (Table 6). Generally, the lightness of all the samples in three kinds of replacement decreased with increasing the percentage of replacement. Decreasing the lightness of samples due to replacing with sesame and peanut meal milk compared with control sample, as well as decreasing the lightness with increasing the percentage of replacement, was probably due to the darker color of meal milk used in mayonnaise compared with egg. Furthermore, decreasing the lightness of samples with increasing the replacement percentage can be due to the increase in the diameter of the particles’ size and the decrease in light segregation (Mcclements & Demetriades, 1998). In examining the obtained results in relation to factor a*(tendency to redness), the difference between the treatments in each day of the period was significant (*p* < .05) (Table 6). The range of the negative numbers observed in the control sample and for 25% and 50% in all the three kinds of replacements in the day after production showed that this negative range suggests a tendency to the samples’ greenness. For the sample S25, this range was negative until the last day of storage (60th day). The control sample in the day after production had the most tendency to greenness compared with other samples, but in examining the general trend of greenness index during the period, the most tendency belonged to the sample S25. The P‐S75 and 100, P100, and S100 had a positive range over the period of storage, which indicated a tendency to redness in these samples during 2 months of storage. Based on factor b* (tendency to yellowness), the difference between the treatments in each time of the period was significant (*p* < .05) and the highest tendency to yellowness (b*) over the period of storage, except the 20th day, was related to the control sample. However, in the 20th day, the highest b* belonged to the sample S25, and statistically, a significant difference was observed among this sample and the control sample. Until the 20th day of storage, the 75% samples of peanut and sesame had the lowest tendency to yellowness. However, in the 40th day, the sample of P‐S100, and in the 60th day, the lowest tendency to yellowness belonged to the samples of P‐S75 and 100 and S100 (Table 6). By increasing the replacement percentage for all three kinds of replaced materials, the tendency to yellowness decreased. Because of that, the color of egg yolk is attributed to yellowish fat‐soluble carotenoids, that is, xanthophylls, including lutein, zeaxanthin, β‐cryptoxanthin, and minor amounts of β‐carotene (Laca, Saenz, Paredes, & Diaz, 2010). In total color change (Δ*Ε*) of mayonnaise samples between the first day and the 60th as a function of storage time, the highest amount of Δ*Ε* was for the sample P‐S75 and the lowest belonged to the control sample, which indicates the stability of color in the control sample over the 2‐month period. Samples P‐S75 and S100, in contrast to other samples, had a significant difference in the control sample (Table 2).

**Table 6:** Variation of the mayonnaise L*, a*, and b* (in all replacement levels of egg yolk with meal milk) at the first and 60th production day in storage temperature(25 ± 1°C)

Treatment	L* (1st→60th) day		a* (1st→60th) day		b* (1st→60th) day	
Blank	88.93 ± 1.05^a^	86.08 ± 2.18^a^	−0.88 ± 0.37^c^	0.08 ± 0.89^def^	28.02 ± 0.5^a^	28.41 ± 0.43^a^
25 Peanut–Sesame	89.28 ± 1.66^a^	86.17 ± 0.93^a^	−0.6 ± 0.71^bc^	0.27 ± 1.14^bcdef^	27.05 ± 0.44^ab^	25.92 ± 0.9 ^cd^
50 Peanut–Sesame	88.2 ± 0.76^a^	84.81 ± 1.07^ab^	0.56 ± 0.32^abc^	0.16 ± 0.11^cdef^	24.32 ± 1.17^d^	26.79 ± 0.37^bc^
75 Peanut–Sesame	87.91 ± 1.43^a^	82.95 ± 1.24^bc^	−0.12 ± 0.08^ab^	1.26 ± 0.18^b^	23.29 ± 0.25^de^	24.1 ± 0.69^e^
100 Peanut–Sesame	87.7 ± 1.27^b^	81.69 ± 2.66 ^cd^	0.51 ± 0.35^a^	1.2 ± 0.16^bc^	23.65 ± 0.36^de^	23.17 ± 0.79^e^
25 Peanut	88.35 ± 0.7^a^	85.96 ± 0.43^a^	0.2 ± 0.57^abc^	0.07 ± 1.05^def^	25.79 ± 0.66^c^	26.56 ± 0.82^bc^
50 Peanut	87.17 ± 2.34^ab^	84.45 ± 0.95^ab^	−0.15 ± 0.73^abc^	0.62 ± 0.43^bcde^	25.56 ± 0.85^c^	26.69 ± 0.34^bc^
75 Peanut	86.36 ± 1.63^ab^	82.73 ± 0.8^bc^	−0.51 ± 0.68^bc^	1.29 ± 0.32^b^	23.06 ± 0.42^e^	24.28 ± 1.48^e^
100 Peanut	79.45 ± 1.56^c^	79.78 ± 1.54^de^	0.62 ± 0.66^a^	2.84 ± 0.17^a^	21.02 ± 0.55^f^	23.5 ± 1.43^e^
25 Sesame	87.98 ± 1.22^a^	85.21 ± 1.57^a^	−0.47 ± 0.53^bc^	−0.27 ± 1.3^ef^	28.07 ± 0.19^a^	27.85 ± 1.39^ab^
50 Sesame	87.45 ± 2.11^ab^	86.08 ± 0.8^a^	−0.87 ± 00.35^c^	−0.77 ± 0.52^f^	26.04 ± 0.8^bc^	26.21 ± 0.53^c^
75 Sesame	87.08 ± 1.21^ab^	85.78 ± 0.61^a^	0.21 ± 0.28^ab^	−0.04 ± 0.63^ef^	23.03 ± 0.51^e^	24.59 ± 1.13^de^
100 Sesame	81.2 ± 2.53^c^	87.34 ± 0.33^e^	0.12 ± 0.18^ab^	1.08 ± 0.3^bcd^	24.38 ± 0.83^d^	23.48 ± 0.47^e^

Assays were performed in triplicate. Mean ± SD values, followed by the same superscript letter within each column (day), have no significant differences in *p *≤ .05 by ANOVA.

### 3.5 Rheological properties of mayonnaise

#### 3.5.1 Flow curves

The rheological models mainly used to test the fitness of the flow behavior of mayonnaise are the power law model (Maruyama, Sakashita, Hagura, & Suzuki, 2007; Worrasinchai, Suphantharika, Pinkai, & Jamnong, 2006) and Herschel–Bulkley model (Bligh & Dyer, 1959; Su, Lien, Lee, & Ho, 2010); these two models have also been used in this research. In regard to the high explanation coefficients of data related to the power law model (95%–99%) shown in Table 7, this model shows suitable conformity to the flow behavior of samples. In the power law model, the flowing index amount (*n*) of all samples is in the domain between zero and one, which is evidence of pseudoplastic behavior (shear thinning) of mayonnaise samples. Among all substituted samples, the conventional sample (Blank) had the highest consistency coefficient, while P‐S75 had the lowest consistency coefficient and the highest flow index. The Herschel–Bulkley model is commonly used for describing the non‐Newtonian fluid flow behavior with a yield stress, where the yield stress is the minimum necessary shear stress needed to start the flow. The parameters related to the Herschel–Bulkley model are mentioned in Table 7. Generally, the yield stress is reduced by increasing the substitution percent, except in the case of P‐S100 and S75, which showed different behavior and had a higher yield stress than samples with lower substitution percent than themselves; of course, the difference amount is low. The most and least amount of yield stress was related to the S25 and P100 samples, respectively.

**Table 7:** Parameters related to power law and Herschel–Bulkley law for different mayonnaise treatments

Treatment	Power Law			Herschel–Bulkley Law			
	k(Pa·s^n^)	n(–)	*R* ^2^	Yield stress (Pa)	k(Pa·s^n^)	n(–)	*R* ^2^
Blank	246.69	0.12	.95	69.31	321.96	0.1	.97
Peanut–sesame 25	174.1	0.13	.97	74.40	92.42	0.21	.98
Peanut–sesame 50	173.93	0.12	.97	48.43	122.94	0.15	.97
Peanut–sesame 75	69.21	0.19	.99	18.41	47.92	0.25	.99
Peanut–sesame 100	117.7	0.11	.97	65.37	45.84	0.23	.99
Peanut 25	191.52	0.13	.97	23.87	167.36	0.15	.98
Peanut 50	160.63	0.13	.98	15.87	144.57	0.14	.98
Peanut 75	123.5	0.13	.98	13.53	109.59	0.14	.99
Peanut 100	121.76	0.14	.98	9.48	112.20	0.14	.99
Sesame 25	199.97	0.12	.97	75.39	118.61	0.18	.97

Overall, the results showed that in the mayonnaise produced by sesame and peanut meal milk and a mixture of them as replacements for egg, the significant physicochemical features in the product shelf‐life were maintained. In addition, according to power law model all of the treatment showed pseudoplastic behavior (dilatant with shear). Also, all the samples were desirable in terms of stability, especially the 100% replaced samples (without egg), which indicated the highest levels of both physical stability and thermal stability. Of course, with regard to the obtained results of the colorimetry data, that is, the decrease in the lightness of the samples with the high percentage of replacement, it is likely for the product not to be welcomed warmly on the part of the consumers. About this, we need to justify the consumers. The positive results of this research are employing suitably sesame and peanut meal milk and a mixture of them instead of egg yolk, decreasing the cholesterol of mayonnaise and increasing its nutritional value, preparing the way for using the meal of the oil extraction factory as emulsifier in mayonnaise factories, which lead to a decrease in overall costs of producing these products.

## ETHICAL STATEMENTS

The authors declare that they do not have any conflict of interest, and the study does not involve any human or animal testing.
